# A System for Recording Dental Operations by Indicators

**Published:** 1850-01

**Authors:** F. L. Crane

**Affiliations:** Easton, Pa.


### ARTICLE II.

*A System for Recording Dental Operations by Indicators.* By F. L. CRANE, D. D. S., *Easton, Pa.*

To be able to turn to a record of past operations, has frequently, and for various reasons, been to me a source of gratification. A system for keeping dental accounts, by which such a record is made with facility and accuracy, is at least desirable. I submit the following sketch of a system to my brother dentists, that it may be compared with others and improved. As the indicators cannot be presented to the eye in this article, they must be supplied by pen or pencil during its perusal.

In order to understand the following details the more practically, take the blank leaf of a day book, and rule three spaces, each a quarter of an inch wide, parallel with, and closely to the left of those which are ruled for the reception of cash amounts. The first, or left space, is for the reception of the division indicators; the second for the class; the third for the surface of the tooth operated upon.

The thirty-two teeth are considered in four divisions, of eight teeth in each, the median line being the line of division, called the upper right, upper left, lower right and lower left divisions. To indicate in which of these divisions the particular tooth operated upon is situated, a short perpendicular line is made in the first or division space, with a short horizontal line at the top and right of the same, for the upper right division; top and left, for the upper left; bottom and right, for the lower right, and bottom and left, for the lower left division: a small right angular figure.

The teeth, for the sake of convenience, are reckoned in eight classes, of four teeth in each class. One letter placed in the second space, and following the angular figure, will indicate the particular tooth in the division you wish to record: thus *f* stands for front incisor; *l*, for lateral incisor; *c*, cuspidatus; *b*, first bicuspid; *d*, second bicuspid; *m*, first molar; *o*, second molar; *r*, third molar.

To show the surface operated upon, one letter also follows, placed in the third space. Thus *m*, in this space, stands for mesial surface, (anterior surface of the molares and bicuspides;) *a*, antimesial surface, (posterior surface of the molares and bicuspides, and right lateral surfaces of the incisores of the right divisions, and left lateral surfaces of the incisores of the left divisions;) *e*, external, (labial,) surface; *i*, internal, (lingual,) surface; *g*, grinding surface.

These names for the surfaces, it is believed, will be found to be more convenient and definite than any others; *anterior* and *posterior* are not sufficiently definite for all of the teeth, being situated in the form of an arch; and *labial* and *lingual* have the same initials.

The signs to indicate the operations performed are placed upon the left line, immediately preceding the division indicator, which line should be ruled double. A small dot or period, placed upon this line, stands for the word *filled;* two dots, *refilled;* and a short dash across the line shows that the given surface has been filed and polished without being filled. The sign for filling may be varied to any required extent, to show the position of a filling upon a given surface; thus, for a posterior depression upon a grinding surface, use a common check mark, carrying the pen to the right upward, at an angle of forty-five degrees, and for an anterior depression use a similar mark, except that the tail is carried to the left. A comma indicates a filling near the external surface; and the same character, inverted, records a filling near the internal or lingual surface. These latter signs are principally used for the grinding surfaces when the filling is not central.

These sets of indicators, in their proper connection, are convenient to use in writing out the details of any case in which particular teeth, &c., are to be designated, as they save time and space.

More than half of these symbols may be dispensed with in recording a series of operations in the same mouth, and often one only is necessary after the first operation is on record, which, it will be perceived, requires four indicators; for, as each operation is recorded beneath the preceding one, it is unnecessary to repeat the sign for filling, until a different symbol is required; nor need the division indicator be repeated as long as you are recording operations in that particular division; or if the division be changed for another, and the class or surface the same, it is not necessary to repeat their indicators, or place one letter or symbol immediately beneath another similar one. The propriety of this will be quickly perceived by trying a few examples upon paper. I think it well to carry out the price received or charged for each operation, on the line with its record, for filing, &c., as well as for filling. I know some who charge little or nothing for filing, whereas it is an operation in which their reputation is perhaps as much concerned as in any other; and I would ask whether it is not reasonable and fair that they should receive at least half as much for preserving a tooth by filing and polishing, as they would have asked for filling the same with gold. A sufficient alphabet should be bound in the case book, in which to enter every patient's name, whether the operations be paid for or not; if paid, they should be so marked in plain letters; if not, they stand charged. I think it best to give the bookbinder directions to rule each page of the case-book for two headings, and put two names upon a page, those being at the top. The record will leave a blank space by its side for remarks, for which it is convenient to make use of short hand or phonography. To such as may not have paid attention to this elegant style of reporting, I will state, that the "Complete Phonographic Class Book, containing an Inductive Exposition of Phonography," may be obtained by mail, of John F. Trow, 49 and 51 Ann-st., New York, price 37½ cents.

